# Effect of Lanthanum Addition on Formation Behaviors of Inclusions in Q355B Weathering Steel

**DOI:** 10.3390/ma15227952

**Published:** 2022-11-10

**Authors:** Ning Mao, Wensheng Yang, Dehong Chen, Wenli Lu, Xiaowei Zhang, Shiying Chen, Minglei Xu, Bo Pan, Liguo Han, Xiaoqiang Zhang, Zhiqiang Wang

**Affiliations:** 1 GRIREM Advanced Materials Co., Ltd., Beijing 100088, China; 2 National Engineering Research Center for Rare Earth, GRINM Group Co., Ltd., Beijing 100088, China; 3 GRIREM Hi-Tech Co., Ltd., Langfang 065201, China

**Keywords:** lanthanum, Q355B weathering steel, nonmetallic inclusion, thermodynamic calculations

## Abstract

The effect of lanthanum addition on the formation behaviors of inclusions in Q355B weathering steel was investigated by laboratory experiments and thermodynamic calculations. The results demonstrate that the main inclusions in weathering steel without La addition are large-sized irregular Al_2_O_3_ and MnS, with an average size of about 5.35 μm. As La content increases from 0.0075 to 0.0184 wt.%, the dominant inclusions transform from MnS, LaAlO_3_, and Al_2_O_3_-LaAlO_3_ into MnS, La_2_O_3_, and LaAlO_3_-La_2_O_3_. Meanwhile, the average size of inclusions significantly decreases from 3.4 to 2.48 μm and the distribution is more dispersive. When the La content increases to 0.0425 wt.%, the original MnS and Al_2_O_3_ inclusions are completely modified into La_2_O_2_S and La_2_O_3_ but the inclusions demonstrate serious agglomeration and growth. The thermodynamic calculations indicate that Al_2_O_3_ and various lanthanum-containing inclusions are formed in the liquid phase. As the La content in molten steel increases from 0 to 0.0425 wt.%, the Al_2_O_3_ inclusion is inclined to be modified into lanthanum oxide and lanthanum oxysulfide and the modification process is Al_2_O_3_ → LaAlO_3_ → La_2_O_3_ → La_2_O_2_S, which is very consistent with the experimental observations.

## 1. Introduction

Weathering steel contains up to 2 wt.% of Cu, P, Cr, Ni, and other alloying elements. Compared with plain carbon steel, weathering steel has excellent atmospheric corrosion resistance due to the addition of these alloying elements, which forms a protective rust layer during atmospheric corrosion. The rust layer consists of a loose outer rust layer and a dense inner rust layer. The outer rust layer is usually composed of α-FeOOH, γ-FeOOH, and Fe_3_O_4_ phase. The inner rust layer is usually composed of extremely dense amorphous spinel Fe_3_O_4_ and α-FeOOH phase. The contact area between the internal rust layer and the matrix is highly smooth, and Cu, P, and Cr are uniformly enriched in the amorphous oxide layer. The amorphous spinel oxide layer adjacent to the matrix reduces the number of anodic sites and hinders the penetration of corrosion particles into the matrix, thus effectively improving the corrosion resistance [1,2,3]. Therefore, it is widely used in container plates, railway vehicles, bridge engineering, and other fields [4,5]. Nowadays, in addition to excellent corrosion resistance, weathering steel is required to possess outstanding mechanical properties, such as strength and toughness, to meet increasingly stringent application requirements. Consequently, improving comprehensive properties, including the corrosion resistance, strength, and toughness on the basis of cost control, is the future direction of weathering steel.

Nonmetallic inclusions are frequently cited as the cause of steel performance issues. They not only deteriorate the corrosion resistance but adversely affect the mechanical properties. The pitting corrosion is mainly caused by manganese sulfide (MnS), deformed silicomanate, and aluminum oxide (Al_2_O_3_), which are not conducive to the formation of a uniform rust layer on the substrate and reduce the adhesion between the rust layer and the matrix, thus resulting in the rust layer falling off [6,7,8,9]. In addition, due to the difference in thermal expansion coefficients and deformation capacity between the inclusions and matrix, stress concentration is prone to occur around the inclusions under load, which leads to the initiation of fatigue cracks [10,11,12].

The addition of rare earth elements can not only purify molten steel but modify inclusions and act as microalloying elements because of their unique electronic layer structure, variable valence, and large-sized atoms. Therefore, adding an appropriate amount of rare earth to steel can significantly improve its corrosion resistance and mechanical properties (e.g., strength, toughness, and heat ductility) [13,14,15,16]. At the same time, it is worth mentioning that, compared with the alloying elements Cr, Ni, and Cu in weathering steel, the price of light rare earth metals is lower. Accordingly, it is undoubtedly an excellent way to improve the comprehensive performance of weathering steel by adding an appropriate amount of light rare earth elements. In recent years, there have been many reports on the research of rare earth weathering steel. Xintong Lian’s [17] research on rare earth weathering steel showed that the grain refinement effect of rare earth (Ce and La) increased the density of the protective rust layers and the formation of RE inclusions also weakened the micro-area electrochemical corrosion caused by MnS inclusions. In addition, the segregation of trace rare earth elements at the interface between the corrosion layers and the matrix enhanced the compactness and adhesion of the rust layers and prevented contact of corrosive ions with the matrix. Therefore, the addition of rare earth obviously improved the corrosion resistance of weathering steel. L J Yue [18] showed that by adding an appropriate amount of rare earth into the weathering steels, the strip-shaped MnS inclusions were modified into the dispersive spherical rare earth inclusions. Moreover, the grain refinement and solid solution strengthening effect of rare earth significantly improved the impact toughness and strength of weathering steel.

Plenty of researchers have reported the effect of rare earth in weathering steel, revealing the effects of inclusion modification, molten steel purification, and microalloying on the mechanical properties and corrosion resistance [16,17,18,19,20,21,22,23]. However, few detailed studies have been performed on the modification mechanism of rare earth on inclusions in weathering steel. Therefore, the modification mechanism of lanthanum on inclusions in Q355B weathering steel was studied by combining laboratory experiments and thermodynamic calculations in this work.

## 2. Materials and Methods

In the present work, four sets of laboratory-scale experiments with different La contents were carried out in a levitation melting furnace. The Q355B weathering steel ingot was loaded into the water-cooled copper crucible of a 1 kg levitation melting furnace. After vacuuming below 10 Pa, high-purity argon gas was added to 50,000 Pa and then gradually heated to 1600 °C for 10 min. Then, La (>99.99 wt.%) wrapped in iron foil was added into the molten steel and then poured into a 70 × 50 × 40 mm mold after holding for 5 min. The ingot was slowly cooled in the furnace after pouring. The addition of La in the four sets of test steels were 0 wt.%, 0.0075 wt.%, 0.0184 wt.%, and 0.0425 wt.%. The final composition of the test steels were determined using ICP-OES (ICPS-8100; Shimadzu corporation, Kyoto, Japan) with an accuracy of ± 0.5 ppm. The accuracy and precision of the component analysis were improved by background subtraction and spectral line selection, and the recovery rate test and accuracy test proved that the method was accurate, sensitive, and reliable. The total oxygen content (T.O) refers to the sum of dissolved oxygen and the oxygen content of the inclusions. It was measured by an ON-3000 oxygen and nitrogen analyzer, which adopted a non-dispersive infrared detection system and was equipped with two independent infrared absorption cells. In addition, the length of the absorption cell and the number of channels could be flexibly adjusted depending on the sample, thus ensuring the sensitivity and reliability of the detection. The chemical composition of each test steel is listed in Table 1. Since the weight fractions of trace non-critical elements, such as Ti, Co, and V, were not included in Table 1, the sum of the weight fractions was less than 100%. To clarify the formation behavior of inclusions in the test steel, four 10 × 10 × 10 mm samples were cut from a 1/2 distance from the center by wire electrode discharge cutting at the same height position of each ingot and then were pre-milled and polished. The chemical composition and morphology of inclusions were analyzed by scanning electron microscopy (SEM) and energy dispersive spectrometer (EDS) attached to an SEM (SU-1500; Hitachi, Ltd., Tokyo, Japan). The selection of the acceleration voltage and the identification of overlapping peaks were carefully processed to ensure the reliability of inclusion analysis in EDS analysis. The image analysis software Image-Pro Plus 6.0 was used to evaluate the size distribution, number density, and average size of inclusions, and at least 50 viewing fields at 2000 magnification were randomly selected in each sample. Additionally, the modification mechanism of lanthanum on the inclusions was analyzed using thermodynamic calculation. The transformation process of inclusions in molten steel with different La contents at 1600 °C was calculated using the thermodynamic software FactSage 8.1 with the databases of FSstel, FactPS, Ftoxid, and FTlite. The FToxid FactSage database could realize the thermodynamic calculation of complex solutions, where the solution system mainly adopted the quasi-chemical model. Compared with the traditional Wagner approximation formula, FactSage software used an “association compound” to characterize the interaction between solute elements, thus ensuring the accurate prediction of the precipitation behavior of complex inclusions in molten steel.

## 3. Results and Discussion

### 3.1. Effect of La Treatment on Characteristics of Inclusions

**Figure 1:**
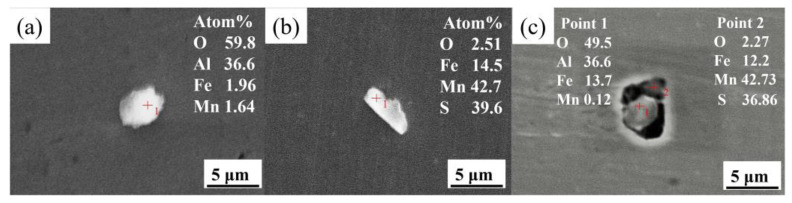
SEM images and EDS results of typical inclusions in RE-free steel: (**a**) Al_2_O_3_, (**b**) MnS, and (**c**) Al_2_O_3_-MnS complex inclusion.

The morphology and chemical composition of typical inclusions in RE-free steel are shown in Figure 1. It can be seen that the inclusions are mainly Al_2_O_3_ and MnS. Additionally, some alumina and manganese sulfide complex inclusions were also found. The core of the complex inclusion is Al_2_O_3_, surrounded by MnS inclusions, as shown in Figure 1c. This is because the solubility of S^2−^ and Mn^2−^ in molten steel decreases with the decrease in solidification temperature during the solidification process [24] and the first-principles calculation of S^2−^, Mn^2+^, and Al_2_O_3_ shows that Al_2_O_3_ has a strong attraction to S^2−^ and Mn^2+^ and can easily form complex inclusions [25]. Therefore, during the solidification process, some small-sized Al_2_O_3_ inclusions are inclined to be pushed to the liquid phase and act as the sites of MnS inclusion formation due to the micro-segregation of Mn and S.

**Figure 2:**
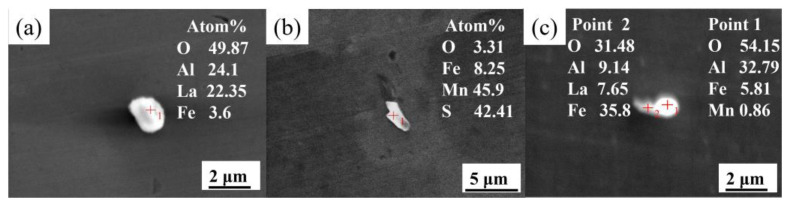
SEM images and EDS results of typical inclusions in steel containing 0.0075 wt.% La: (**a**) LaAlO_3_, (**b**) MnS, and (**c**) Al_2_O_3_-LaAlO_3_ complex inclusion.

Figure 2 displays the typical inclusions in sample steel containing 0.0075 wt.% La. According to the atom fraction of the elemental composition of inclusion, the inclusions are mainly spherical LaAlO_3_ and irregular MnS. Additionally, a small number of irregular Al_2_O_3_ and LaAlO_3_ complex inclusions can also be found. As shown in Figure 2c, its main body is still Al_2_O_3_ and the outer part is modified by La. Consequently, the harmful Al_2_O_3_ and MnS inclusions cannot be completely modified by adding a small amount of La.

**Figure 3:**
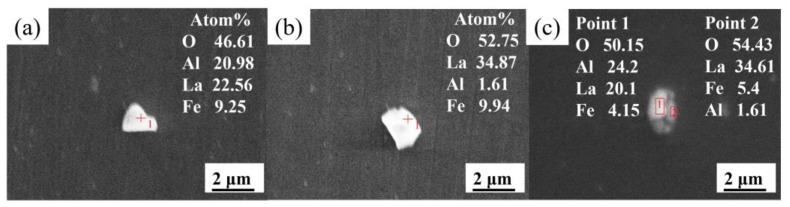
SEM images and EDS results of typical inclusions in steel containing 0.0184 wt.% La: (**a**) LaAlO_3_, (**b**) La_2_O_3_, and (**c**) LaAlO_3_-La_2_O_3_ complex inclusion.

As shown in Figure 3, the Al_2_O_3_ inclusions are completely absent. The inclusions are mainly LaAlO_3_, La_2_O_3_, MnS, and LaAlO_3_-La_2_O_3_ complex inclusions as the La content in steel increases to 0.0184 wt.%.

**Figure 4:**
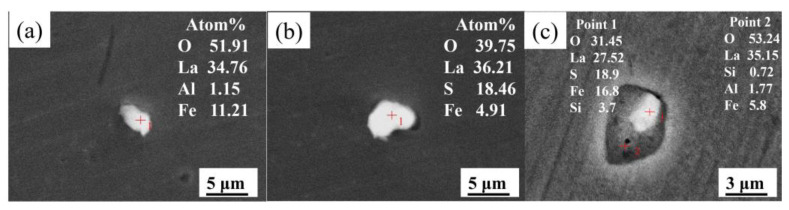
SEM images and EDS results of typical inclusions in steel containing 0.0425 wt.% La: (**a**) La_2_O_3_, (**b**) La_2_O_2_S, and (**c**) La_2_O_2_S-La_2_O_3_ complex inclusion.

As shown in Figure 4, when the La content increases to 0.0425 wt.%, the MnS and Al_2_O_3_ inclusions disappear completely and the inclusions are mainly La_2_O_2_S and La_2_O_3_ and a small amount of La_2_O_2_S-La_2_O_3_ complex inclusions. As shown in Figure 4c, the morphology of complex inclusion is a La_2_O_3_ inclusion wrapped in a La_2_O_2_S inclusion. This is because the La_2_O_3_ inclusions consume the surrounding oxygen during its formation and the formation condition of La_2_O_2_S is first satisfied around La_2_O_3_ inclusions.

**Figure 5:**
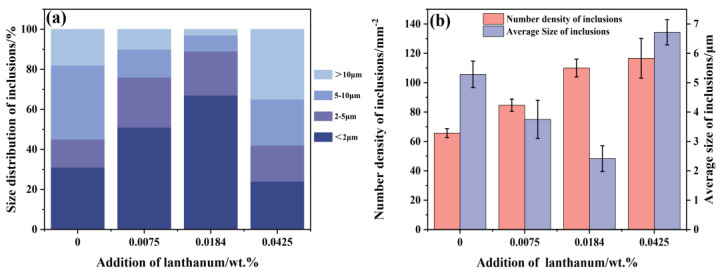
Size distribution (**a**) and number density and average size (**b**) of inclusions in four steels with different La content additions.

To investigate the influence of La treatment on the quantity and size distribution of inclusions, the inclusions were statistically measured using Image-Pro software. The size-distribution statistical data show that the inclusions in RE-free steel are large-sized, which can heavily deteriorate the mechanical performance and corrosion resistance of weathering steel [12,26]. With the increase in La content, the percentage of inclusions larger than 5 μm gradually decreases. However, when the La addition reached 0.0425 wt.%, the proportion of inclusions larger than 10 μm remarkably increases, and inclusions larger than 5 μm are dominant, reaching 65%. Meanwhile, it can be seen in Figure 5b that with the increase in La content, the number density of inclusions continuously increases, and the average size of inclusions gradually decreases. The average size of inclusions in the RE-free steel is 5.35 μm. As the La content increase from 0.0075 to 0.0184 wt.%, the average size of inclusions significantly decreases from 3.4 to 2.48 μm, which means that the inclusions become finer and more dispersive. This is because, compared with Al_2_O_3_ and MnS inclusion, rare earth inclusion has lower interfacial energy and better wettability with liquid steel and thus is not easy to agglomerate [27].

However, as the La content reaches up to 0.0425 wt.%, the average size of inclusions sharply increases to 6.85 μm and even exceeds that of the original ones, which indicated that the inclusions seriously grow and agglomerate. This is because rare earth elements have extremely high surface activity and easily adsorb impurity elements in molten steel. The higher the concentration of rare earth in molten steel, the more impurity elements adsorbed. On the other hand, excessive rare earth promotes mutual attraction between rare earth inclusions and then merges into large-sized inclusions. Therefore, when the addition is excessive, rare earth inclusions are prone to agglomeration and growth. Many investigators also reported this regulation in their work [28,29,30]. In addition, it is worth mentioning that there is an optimum addition amount in the range of 0.0184~0.0425% La, which can significantly reduce inclusion size and improve corrosion resistance and mechanical properties.

The above experimental results indicate that the original Al_2_O_3_ and MnS inclusions can be modified to fine and dispersive rare earth inclusions by adding a suitable amount of La. At the same time, the La content has a great influence on the type of inclusion in steel. The main inclusions in weathering steel without La addition are Al_2_O_3_ and MnS. As the La content increases from 0.0075 to 0.0184 wt.%, the main inclusions change from LaAlO_3_, MnS, and Al_2_O_3_-LaAlO_3_ complex inclusion to LaAlO_3_, La_2_O_3_, MnS, and LaAlO_3_-La_2_O_3_ complex inclusion. When the La content reaches to 0.0425 wt.%, the original MnS and Al_2_O_3_ inclusions are completely modified into La_2_O_2_S and La_2_O_3_ inclusions.

Some similar modification rules have been previously reported in other studies on rare earth steels. Huang Y [31] added Ce into H13 steel and found that with the increase in Ce content, the inclusions were modified according to the rule of MgAl_2_O_4_ → CeAlO_3_ → Ce_2_O_3_. Ren et al. [32] found that when Ce was added to ultra-low carbon steel, the inclusions were modified along the route of MgAl_2_O_4_ → CeAlO_3_ → Ce_2_O_2_S → Ce_2_O_2_S → CeS.

### 3.2. Thermodynamic Analysis on the Formation and Modification of Inclusions

In order to analyze the formation and modification mechanism of inclusions in steel, thermodynamic analysis was performed in the present work, which can also provide theoretical guidance for controlling inclusions in rare earth steel.

According to the thermodynamic theory of inclusion, during the cooling process of molten steel, the solubility of inclusion decreases continuously and when the actual solubility product of the inclusion generating elements is larger than the equilibrium solubility product, the reaction can occur. Therefore, by comparing the actual solubility product and equilibrium solubility product, the possibility and condition for the reaction of inclusion can be obtained.

The liquidus temperature (*T*_L_) and solidus temperature (*T*_S_) of steel can be calculated by Equations (1) and (2) [33]. The calculation results are 1784.4 K and 1733.9 K, respectively.

(1) $$\;\;T_{L}=1809\;-\;83\;\mathrm{wt}.\%\;\left(C\right)\;-\;7.8\;\mathrm{wt}.\%\;\left(\mathrm{Si}\right)\;-\;5\;\mathrm{wt}.\%\;\left(\mathrm{Mn}\right)\;-\;32\;\mathrm{wt}.\%\;\left(P\right)\;-\;31.5\;\mathrm{wt}.\%\;\left(S\right)\;-\;1.5\;\mathrm{wt}.\%\;\left(\mathrm{Cr}\right)\;-\;2\;\mathrm{wt}.\%\;\left(\mathrm{Mo}\right)\;-\;2\;\mathrm{wt}.\%\;\left(V\right)\;-\;3.6\;\mathrm{wt}.\%\;\left(\mathrm{Al}\right)\;-\;18\;\mathrm{wt}.\%\;\left(\mathrm{Ti}\right)\;\;$$

(2) $${\;T}_{S\;}=1809\;-\;344\;\mathrm{wt}.\%\;(C)\;-\;12.3\;\mathrm{wt}.\%\;(\mathrm{Si})\;-\;6.8\;\mathrm{wt}.\%\;(\mathrm{Mn})\;-\;124.5\;\mathrm{wt}.\%\;(P)\;-\;1.4\;\mathrm{wt}.\%\;(\mathrm{Cr})\;-\;183.5\;\mathrm{wt}.\%\;(S)\;-\;4.1\;\mathrm{wt}.\%\;(\mathrm{Al})\;-\;4.3\;\mathrm{wt}.\%\;(\mathrm{Ni})$$

The standard Gibbs free energy change for the formation of various inclusions in molten steel are shown in Table 2. It is widely known that Al_2_O_3_ and MnS have different crystalline types under different pressures, temperatures, and vapor partial pressure conditions. Since the modification behavior of inclusions studied in this work is under the condition of liquid steel at 1600 °C, the crystalline type of Al_2_O_3_ and MnS at this temperature are α-Al_2_O_3_ and α-MnS in the calculation. Moreover, the crystalline types of these compounds are not related to the thermodynamic calculation.

The reaction equation for the $A_{x}B_{y}$ generated by metal element A and nonmetal element B in molten steel is as follows:

(3) $$xA+y{B=A}_{x}B_{y}$$

When Equation (3) reaches equilibrium, Δ*G* = 0. Therefore, the relation between the standard Gibbs free energy *(*Δ*G*^θ^*)* for the reaction and the equilibrium constant (*K*) can be expressed as Equation (4).

(4) $$\ln K=-\frac{\Delta G^{\theta }}{RT}=-C_{2}-\frac{C_{1}}{T}$$

where *R* is the gas constant in J·mol^−1^·K^−1^, *C*_1_ and *C*_2_ are constants, and *T* is the temperature (K).

The equilibrium constant (*K*) can also be expressed as follows:

(5) $$K\;=\frac{\alpha_{A_{x}B_{y}}}{\alpha_{A}^{x}\cdot \alpha_{B}^{y}}=\frac{1}{\;f_{A}^{x}\cdot w{\left(A\right)}^{x}\cdot f_{B}^{y}\cdot w{\left(B\right)}^{y}}$$

where *α*_A_, *α*_B_ and $\alpha_{A_{x}B_{y}}$ are the activity of A, B and the precipitate $A_{x}B_{y}$, respectively; ƒ_i_ is the activity coefficient of element i; and w(i) is the mass fraction of element i in molten steel (wt.%). If these compounds $A_{x}B_{y}$, with a high melting point, are solid in molten steel above 1700 K, assuming pure substance as the standard state, $\alpha_{A_{x}B_{y}}=1.$

Substituting Equation (5) into Equation (4) can result in Equation (6).

(6) $$x\ln f_{A}+y\ln f_{B}+\ln(w{\left(A\right)}^{x}\cdot w{\left(B\right)}^{y})=C_{2}+\frac{C_{1}}{T}$$

Therefore, the equilibrium solubility product of $A_{x}B_{y}$ in liquid steel can be expressed as Equation (7).

(7) $$\ln(w{\left(A\right)}^{x}\cdot w\left(B{)}^{y}\right)=C_{2}+\frac{C_{1}}{T}-x\ln f_{A}-y\ln f_{B}$$

The activity coefficient $f_{i}$ in Equation (7) is related to the interaction coefficient of each element in liquid steel temperature. The thermodynamic behavior of molten steel deviates from Raoult’s law and Henry’s law due to the solvent–solute interaction and solute–solute interaction. The Wagner model proposed the general formula of activity and interaction parameters in a multi-component solution, which became a basic method to calculate the activity coefficient of a multi-component solution and has been widely used [36]. The limitation of the Wagner model is that the data of the second-order interaction coefficient are missing but the activity coefficient calculated by the Wagner model is correct in the trend analysis of thermodynamic behavior and has little effect on reliability. In this work, Q355B weathering steel is a low alloy steel, which means that the mass fraction of each solute element approaches 0 and the mass fraction of solvent approaches 1. Therefore, the second-order interaction coefficients have little effect on the activity coefficient of the solute. The activity coefficients of each solute element can be calculated directly using the Wagner model characterized by the first-order interaction coefficient:

(8) $$\ln f_{i}=2.303\overset{n}{\underset{j=1}{\sum }}e_{i}^{j}w[j]$$

where $e_{i}^{j}$ is the first-order interaction parameter between i and *j* and w[j] is the mass percentage of element j.

The first-order interaction coefficients of various solute elements in liquid steel as shown in Table 3. Substituting the data into Equation (8), the activity coefficients of O, S, Al, Mn, and La in molten steel can be obtained, in which element j contains the main elements of weathering steel Q355, including C, Si, Mn, P, S, O, Cr, Ni, Mo, Cu, Al, and La. The results are listed in Table 4.

Therefore, the logarithm of the equilibrium solubility product of MnS, Al_2_O_3_, La_2_O_3_, La_2_O_2_S, La_2_S_3_, LaS, and LaAlO_3_ in molten steel can be calculated by introducing the activity coefficient of each element in Table 4 into Equation (7).

The equilibrium solubility product of $A_{x}B_{y}$ in liquid steel can be expressed as Equation (9).

(9) $${\mathrm{lnA}}_{x}B_{y}=\ln\frac{a_{A}^{x}a_{B}^{y}}{a_{A_{x}B_{y}}}=\ln\left({\left(w\left(A\right)\times f_{A}\right)}^{x}\times {\left(w\left(\left[B\right]\right)\times f_{B}\right)}^{y}\right)$$

**Figure 6:**
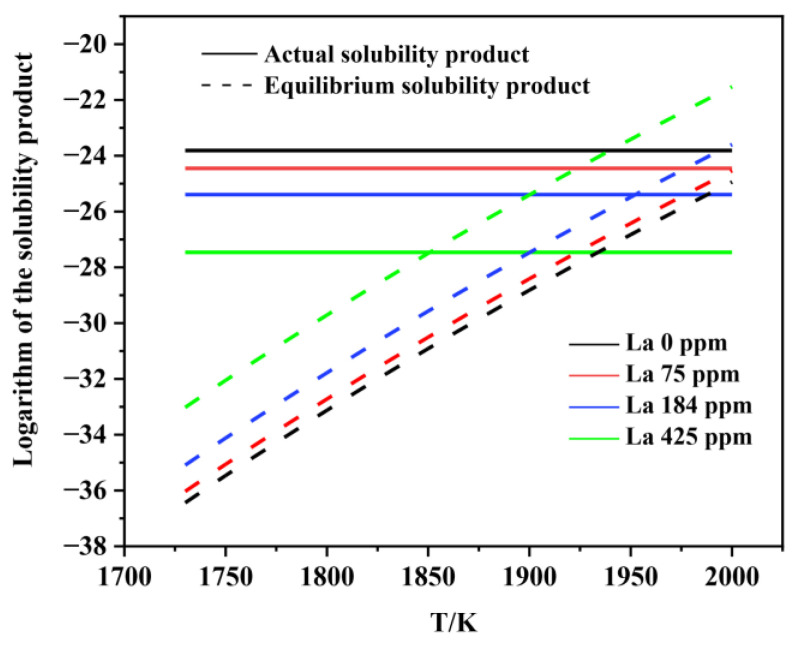
Logarithm of the solubility product as a function of temperature for Al_2_O_3_ in liquid steel.

**Figure 7:**
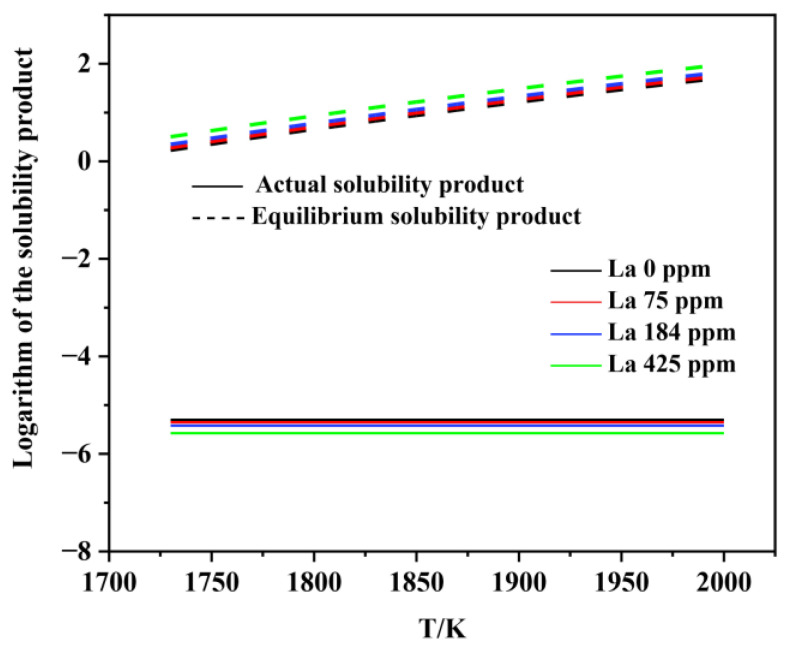
Logarithm of the solubility product as a function of temperature for MnS in liquid steel.

**Figure 8:**
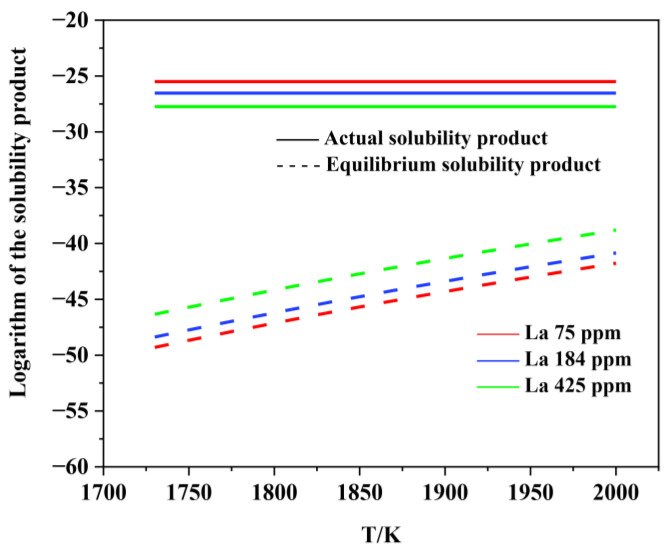
Logarithm of the solubility product as a function of temperature for LaAlO_3_ in liquid steel.

**Figure 9:**
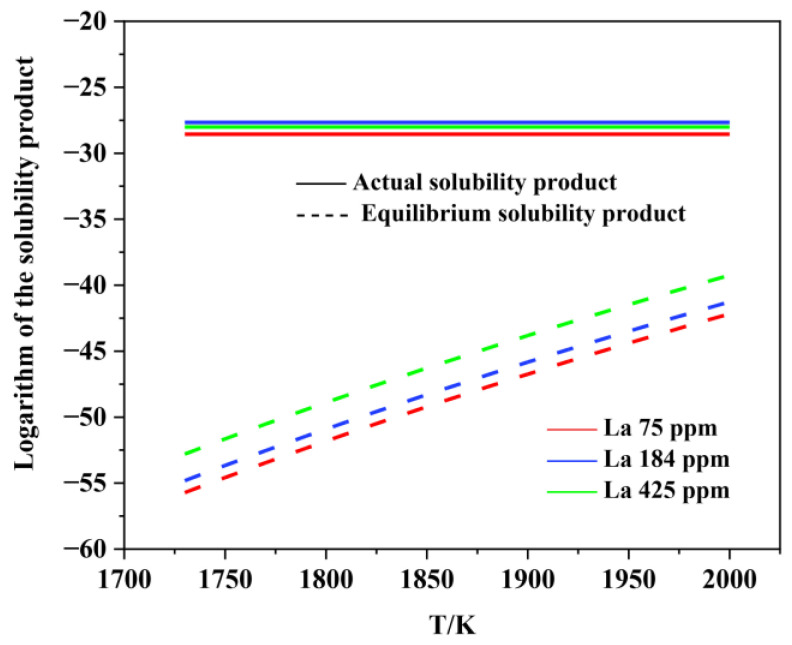
Logarithm of the solubility product as a function of temperature for La_2_O_3_ in liquid steel.

**Figure 10:**
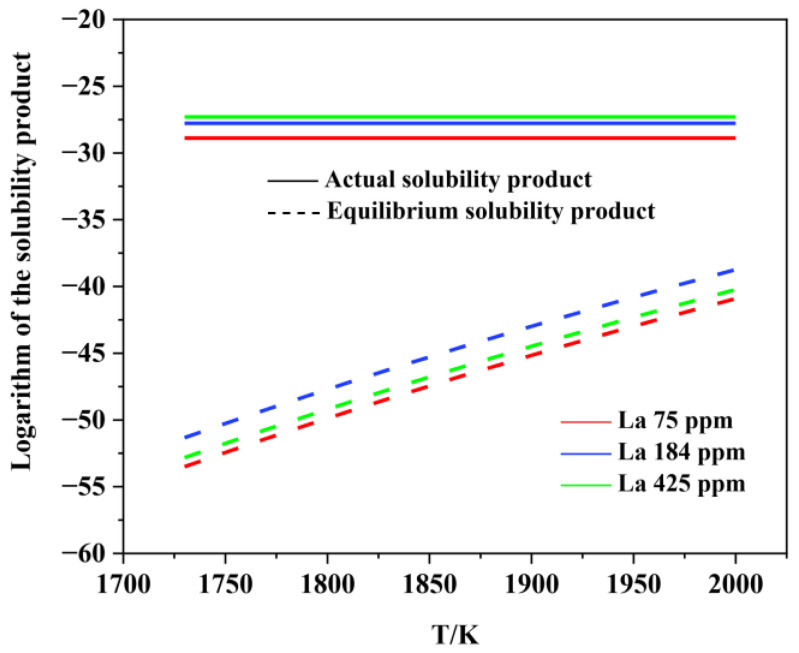
Logarithm of the solubility product as a function of temperature for La_2_O_2_S in liquid steel.

**Figure 11:**
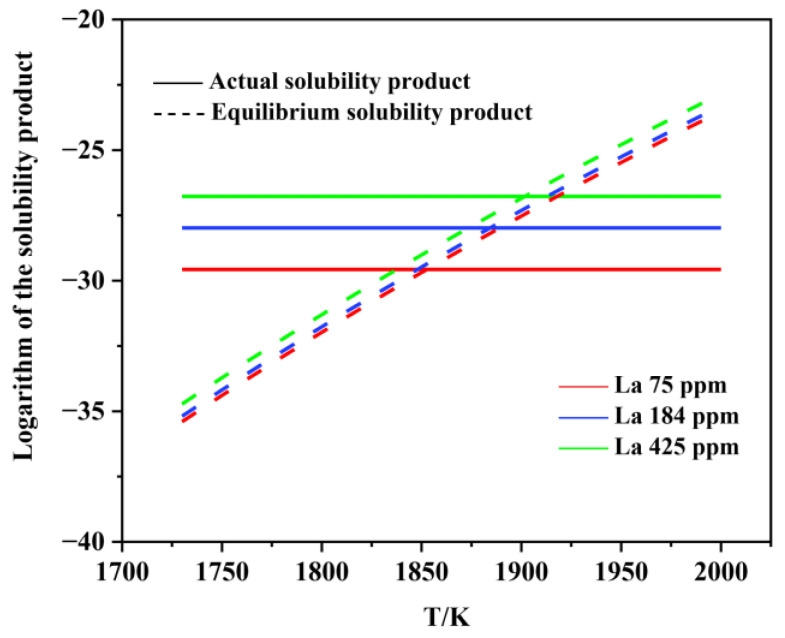
Logarithm of the solubility product as a function of temperature for La_2_S_3_ in liquid steel.

**Figure 12:**
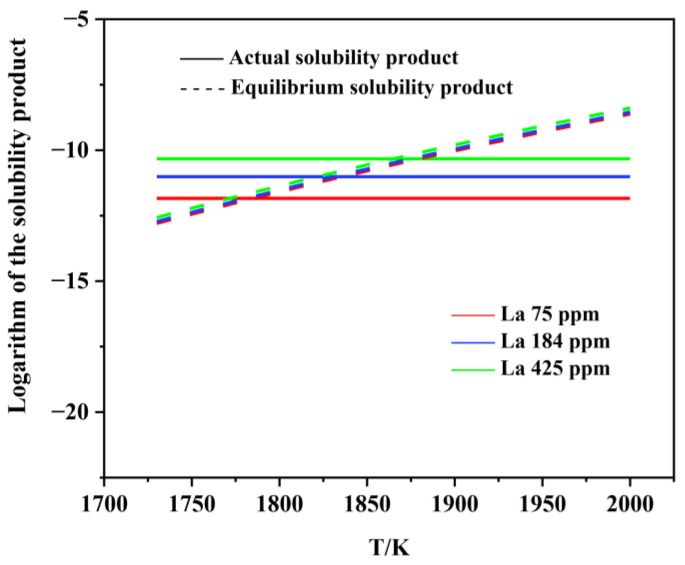
Logarithm of the solubility product as a function of temperature for LaS in liquid steel.

Figure 6, Figure 7, Figure 8, Figure 9, Figure 10, Figure 11 and Figure 12 display the comparison of the equilibrium solubility product and the actual solubility product of each inclusion in liquid steel.

As shown in Figure 6 and Figure 7, Al_2_O_3_ can form at the liquid-phase temperature for each experimental steel. However, the actual solubility product of MnS in each experimental steel was smaller than the equilibrium solubility product, which means that MnS cannot form in the liquid phase. Many previous studies have proven that, during the solidification process, the generating elements of MnS inclusion tend to segregate in the liquid phase at the solidification front, resulting in an increase in the actual solubility product. Therefore, MnS is usually formed in the solidification process, rather than in the liquid phase.

As shown in Figure 8, Figure 9, Figure 10, Figure 11 and Figure 12, when La is added, LaAlO_3_, La_2_O_3_, La_2_O_2_S, LaS, and La_2_S_3_ can form at the liquid-phase temperature, which is because rare earth elements have extremely strong activity and easily react with O and S in molten steel. However, rare earth sulfides are not found in the experimental observations. This is because the formation conditions and sequence of inclusions are also related to the free energy and the mutual transformation between inclusions under actual conditions. Consequently, we further analyzed the stability of various inclusions by calculating the Gibbs formation energy in the molten steel at 1600 °C.

The Gibbs free energy of the inclusion formation reaction (Table 2) in liquid steel can be expressed as:

(10) $$\Delta G_{m}=\Delta G_{m}^{\theta }+RT\ln\frac{a_{{\mathrm{Me}}_{a}X_{b}\left(s\right)}{}^{\frac{1}{a}}}{a_{\left[\mathrm{Me}\right]}\cdot a_{\left[X\right]}{}^{\frac{b}{a}}}$$

where $\Delta G_{m}^{\theta }$ is the standard Gibbs free energy change for the formation of various inclusions, $a_{\left[\mathrm{Me}\right]}$, $a_{\left[X\right]}$ is the activity of each reactant in molten steel, and $\;a_{{\mathrm{Me}}_{a}X_{b}\left(s\right)}$ is the activity of solid inclusion in molten steel. If these inclusions with a high melting point are solid in molten steel at 1600 °C, assuming pure substance as the standard state, $\;a_{{\mathrm{Me}}_{a}X_{b}\left(s\right)}=1.$ The stoichiometric number of La and Al was set to 1 when calculating the actual Gibbs free energy of each inclusion.

Therefore, the Gibbs free energy of the inclusion formation reaction can be simplified as:

(11) $$\Delta Gm=\Delta G_{m}^{\theta }+RT\ln\frac{1}{a_{\left[\mathrm{Me}\right]}\cdot a_{\left[X\right]}{}^{\frac{b}{a}}}$$

The mass concentration of component A in molten steel is w[A] and the activity coefficient is $f_{A}$. The activity of component A can be expressed as Equation (12).

(12) $$a_{A}=f_{A}w\left[A\right]$$

**Figure 13:**
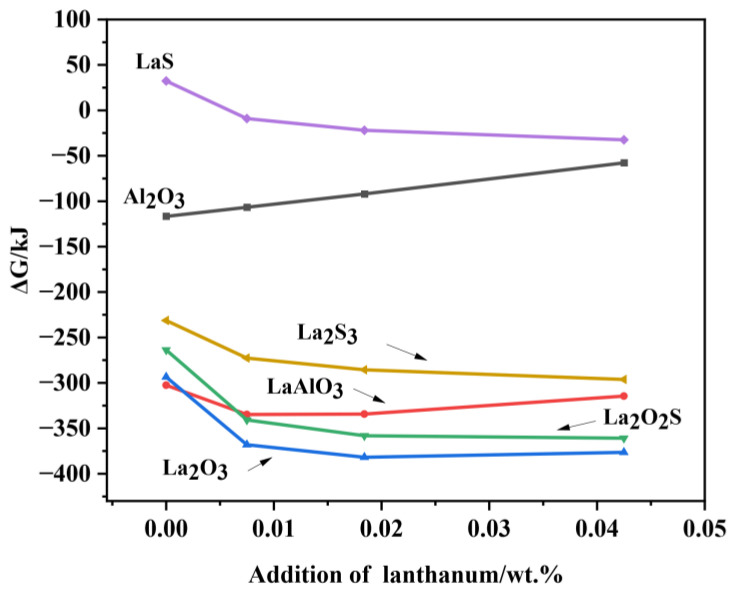
Diagram of inclusion predominance region in steel with different La contents at 1600 °C.

The actual Gibbs free energy of each inclusion formation at 1600 °C is shown in Figure 13. It can be seen that La_2_O_3_ is the most stable, followed by La_2_O_2_S and LaAlO_3_, whose Gibbs generation energy is less than −300 kJ. Therefore, La_2_O_3_, La_2_O_2_S, and LaAlO_3_ inclusions are easier to generate and more stable. Meanwhile, La_2_S_3_, LaS, and Al_2_O_3_ can also form but their Gibbs formation energy is much higher than that of rare earth oxides and rare earth oxysulfide. In addition, it is worth mentioning that with the increase in La content, the Gibbs free energy of the Al_2_O_3_ formation gradually increases, indicating that its formation is more difficult. This is because the rare earth elements have a strong activity, more easily reacting with oxygen in the molten steel, so with the increase in La content, the formation of Al_2_O_3_ is more difficult. Based on the above analysis, it can be determined that with the addition of La, the original Al_2_O_3_ inclusions are more inclined to be modified into La_2_O_3_, La_2_O_2_S, and LaAlO_3_ inclusions.

**Figure 14:**
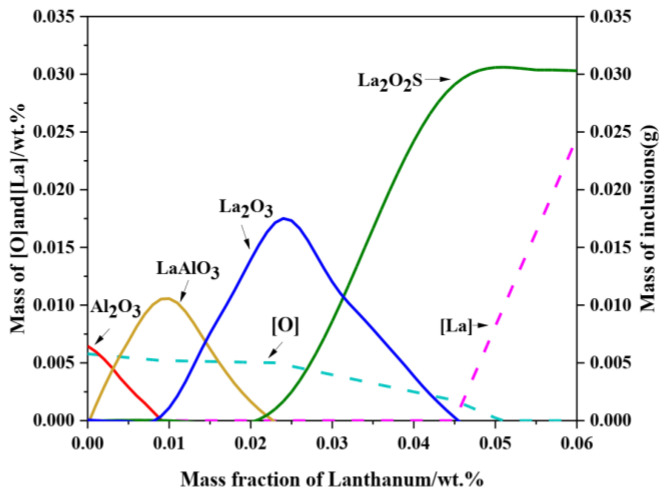
Stability diagram of inclusion predominance region in steel with different La contents at 1600 °C.

To further clarify the effect of La content on the inclusion, the evolution process of La on inclusion at 1600 °C was calculated by FactSage 8.1. The optimized parameters for the binary systems can be used together with appropriate solution models to predict the thermodynamic properties of multicomponent systems, thus calculating phase diagrams of ternary systems. The ternary phase diagram of LaAlO_3_ and La_2_O_2_S can be accurately calculated by combining the phase diagram of the binary systems Al_2_O_3_–La_2_O_3_ and La_2_O_3_–La_2_S_3_ with the calculation model in FactSage software [38,39]. The result is shown in Figure 14. The inclusion in liquid steel without La is Al_2_O_3_. With the increase in La content in liquid steel, Al_2_O_3_ inclusion is immediately modified into LaAlO_3_. When the La content reaches about 0.009 wt.%, the Al_2_O_3_ inclusion disappears completely and the mass fraction of LaAlO_3_ reaches peak value. As the La content increases further, the modified product LaAlO_3_ is transformed into La_2_O_3_. When the La content reaches approximately 0.023 wt.%, LaAlO_3_ is completely turned into La_2_O_3_. Furthermore, it can be seen that, during the formation process of rare earth oxide, the dissolved O in molten steel continuously decreases. Consequently, when the ratio of the oxygen-sulfur concentration reaches a certain amount, La_2_O_3_ is further transformed into La_2_O_2_S with the increase in La content.

Based on the above FactSage thermodynamic calculation results, with the increase in La content, the modification sequence of inclusions in the steel is Al_2_O_3_ → LaAlO_3_ → La_2_O_3_ → La_2_O_2_S, which is very consistent with the experimental observations. Meanwhile, combined with the experimental results, it can be determined that with the increase in La content, the generation of La_2_O_2_S continuously consumes dissolved S in the molten steel, thus inhibiting the formation of MnS at the solidification front. In addition, as can be seen from the dissolved La content in Figure 14, La completely forms inclusions in the molten steel when the La content is low. In contrast, as the La content increases to 0.045 wt.%, the mass fraction of La_2_O_2_S inclusions reaches the maximum and the dissolved La content in the molten steel gradually increases, which can act as a microalloying element.

## 4. Conclusions

Laboratory experiments and thermodynamic analysis were performed to investigate the effect of lanthanum addition on the formation behaviors of inclusions in Q355B weathering steel and the main conclusions obtained are as follows:

1. The original harmful inclusions in Q355B weathering steel could be modified to fine and dispersive rare earth inclusions by adding the appropriate amount of La. The main inclusions in RE-free steel are large-sized irregular Al_2_O_3_ and MnS, with an average size of about 5.35 μm. As the La content increases from 0.0075 to 0.0184 wt.%, the main inclusions transform from MnS, LaAlO_3_, and Al_2_O_3_-LaAlO_3_ complex inclusion into LaAlO_3_, La_2_O_3_, MnS, and LaAlO3-La_2_O_3_ complex inclusion. Meanwhile, the average size of inclusions significantly decreases from 3.4 to 2.48 μm and the distribution is more dispersive. When the La content increases to 0.0425 wt.%, the original MnS and Al_2_O_3_ inclusions are completely modified into La_2_O_2_S and La_2_O_3_ but the inclusions demonstrate serious agglomeration and growth.
2. The thermodynamic calculations indicate that Al_2_O_3_ and various lanthanum-containing inclusions are formed in the liquid phase. As the La content in molten steel increases from 0 to 0.0425wt.%, the Al_2_O_3_ inclusion is inclined to be modified into lanthanum oxide and lanthanum oxysulfide. The modification process is Al_2_O_3_ → LaAlO_3_ → La_2_O_3_ → La_2_O_2_S, which is very consistent with the experimental observations.

## Figures and Tables

**Table 1:** Chemical composition of Q355B weathering steels with different lanthanum contents (wt.%).

Sample	T.O	C	Al	Si	Mn	S	Cr	La
NO. 1	0.0076	0.1740	0.0179	0.1346	1.56	0.0030	0.0549	-
NO. 2	0.0076	0.1611	0.0203	0.1327	1.47	0.0028	0.0566	0.0075
NO. 3	0.0076	0.1657	0.0192	0.1344	1.47	0.0023	0.0555	0.0184
NO. 4	0.0076	0.1791	0.0325	0.2079	1.59	0.0022	0.0554	0.0425

**Table 2:** The standard Gibbs free energy change for formation of various inclusions [34,35].

Reaction Equation of Various Inclusions	$\Delta G^{\theta }=A+B\;\times \;T\;(J\cdot {\mathrm{mol}}^{-1})$	
	A	B
[Mn]+[S]=MnS(s)	−158,365	93.966
$2\left[\mathrm{Al}\right]+3\left[O\right]{=\mathrm{Al}}_{2}O_{3}\left(s\right)$	−122,500	393.8
$2\left[\mathrm{La}\right]+3\left[O\right]{=\mathrm{La}}_{2}O_{3}\left(s\right)$	−1,443,880	337
$2\left[\mathrm{La}\right]+2\left[O\right]+\left[S\right]{=\mathrm{La}}_{2}O_{2}S(s)$	−1,341,200	301
[La]+[S]=LaS(s)	−445,180	141.5
$\left[\mathrm{La}\right]+\left[\mathrm{Al}\right]+3\left[O\right]{=\mathrm{LaAlO}}_{3}\left(s\right)$	−801,616	28.9

**Table 3:** First-order interaction coefficients $e_{i}^{j}$ of various elements in liquid steel at 1600 °C [34,35,37].

$e_{i}^{j}$	C	Si	Mn	P	S	O	Cr	Ni	Mo	Cu	Al	La
O	−0.45	−0.131	−0.021	0.07	−0.133	−0.2	−0.04	0.006	0.0035	−0.013	−3.9	−12.1
S	0.110	0.063	−0.026	0.029	−0.028	−0.27	−0.011	0	0.0027	−0.0084	0.035	−2.79
Al	0.091	0.0056	0.056	0.033	0.03	−6.6	0.025	0.008	-	0.008	0.045	−0.511
Mn	−0.07	0.39	0	−0.0035	−0.048	−0.083	0.003	−0.007	0.0045	-	0.07	-
La	−0.279	-	0.28	1.734	−12.130	−105	-	-	-	-	−2.649	−0.013

**Table 4:** The activity coefficients of O, S, Al, Mn, and La in molten steel at 1600 °C.

No.	O	S	Al	Mn	La
1	0.6290	0.9668	1.140	1.0981	-
2	0.5103	0.9214	1.131	1.098	0.3484
3	0.3764	0.8589	1.116	1.0982	0.3483
4	0.1924	0.7358	1.085	1.0982	0.3480

## Data Availability

Not applicable.
